# Molecular Research on Heart Protection

**DOI:** 10.3390/ijms25010011

**Published:** 2023-12-19

**Authors:** Eltyeb Abdelwahid, Katherine Athayde Teixeira de Carvalho

**Affiliations:** 1 Feinberg Cardiovascular Research Institute, Feinberg School of Medicine, Northwestern University, Chicago, IL 60611, USA; 2 Department of Medicine, University of Illinois at Chicago, 909 South Wolcott Avenue, Chicago, IL 60612, USA; 3 Advanced Therapy and Cellular Biotechnology in Regenerative Medicine Department, Pelé Pequeno Príncipe Institute, Child and Adolescent Health Research & Pequeno Príncipe Faculties, Ave. Silva Jardim, n°1632, Curitiba 80240-020, Paraná, Brazil; katherinecarv@gmail.com

Recently, various molecular bases of heart protection have been discovered and used in many experimental and clinical investigations [1,2,3]. Protective strategies based on molecular research can positively impact a diseased heart by facilitating diagnosis and treatment of injuries and disorders [4,5,6,7]. In this Special Issue, we present original research papers and updated reviews that elucidate molecular mechanisms and potential therapeutic opportunities for heart protection. These investigations further our knowledge of cardiac pathologies and related therapies.

Szponar et al. (2023) generated a model of chronic anthracycline-induced cardiomyopathy [1]. They conducted functional, morphological, biochemical, and molecular studies to examine if combining DEX and CVD pretreatments can attenuate ANT-induced cardiomyopathy. They found no synergy between carvedilol and the preventive effect of dexrazoxane. Tejedor et al. made a genetically modified mesenchymal stem cell (MSC) cell line that releases SEVs expressing functional OSM proteins on their surface to enhance cardiac tissue restoration [8]. The results may contribute to developing of refined MSC-SEV-related treatments for heart tissue restoration. Zanotti et al. explored the effects of sEVs released from DFO-treated ASCs (DFO-sEVs) on human umbilical vein endothelial cells (HUVECs). The authors used transcriptome sequencing and miRNA profiling of the sEV cargo (sEV-miRNA) of treated HUVECs and found significant enhancement of mitochondrial oxidative phosphorylation [9]. 

The results contribute to understanding the mechanisms of cell proliferation and angiogenesis. Pu et al. identified an essential role of Nr1d1 and associated mechanisms in cellular senescence and cardiac aging. Elevated expression of Nr1d1 in cardiac-derived Sca-1+CD31− cells and MCM leads to cell cycle arrest in the G0/G1 phase, suppresses cell proliferation, and promotes apoptotic cell death and senescence [10]. They also silenced Nr1d1 expression in Sca-1+CD31− cells and MCM and found increased cell proliferation and reduced cellular senescence. Nr1d1 appears to cause cellular senescence via inducing Serpina3 expression. The results provide a good basis for therapies targeting heart problems of old age. Ahmad et al. analyzed the effects of MSC injection on post-infarction arrhythmia in a murine double infarction model. MSC treatment after double infarction significantly reduced simple and complex arrhythmias and inhibited inflammatory pathways [11]. The authors were able to uncover changes in the expression of genes that are thought to regulate arrhythmogenesis after myocardial infarction. Gaebe et al. addressed the association between obesity, exercise, and cardiovascular problems by discussing lessons from stem-cell transplantation and preventive exercise. The application of cutting-edge technologies should enable the discovery of targeted interventions for individual risk factors. Future research could identify mechanisms for biomarkers that represent the effect of personalized lifestyle interventions designed for individual risk factors [12]. Wang et al. discussed the optimal conditions for modRNA-based therapy via gene modification strategies and the selection of reliable delivery materials. The authors also discussed the potential effects and mechanisms of modRNA in myocardial infarction as well as the current challenges of modRNA-based heart therapies [13]. It is hoped that future clinical trials will be conducted to advance the application of modRNA in the cardiac field. Höving et al. (2022) reviewed the current knowledge regarding the protective effect of young blood on mammalian organs, including the heart [14]. The authors reviewed essential studies from the murine system, discussed previously reported blood-borne factors and the related signaling mechanisms, and highlighted the respective identification methods identification. The paper summarized clinical trials aimed at utilizing young blood-derived therapies. Overall, there are promising results regarding the using of young blood or young blood-derived factors to rescue age-related pathologies in diverse murine organs, including the heart. These findings await future research to identify the underlying molecular pathways and the potential therapeutic targets.

Taken together, this Special Issue covers a broad span of both original and review papers concerning molecular research on heart protection. The published papers address various regulatory mechanisms and the potential to improve treatments that attenuate the phenotype of patients with cardiomyopathies. These works open new avenues for advancing diagnostics and treatment strategies to improve cardiac function. Particular attention should be given to integrating of various emerging molecular events with networks known to impact cardiac pathophysiology. Advancement of genetic research dissecting the basis of heart diseases would boost the discovery of novel targets for cardiac therapies.
